# Transmission of Viruses from Restroom Use: A Quantitative Microbial Risk Assessment

**DOI:** 10.1007/s12560-023-09580-1

**Published:** 2024-02-19

**Authors:** Sarah E. Abney, Ciara A. Higham, Amanda M. Wilson, M. Khalid Ijaz, Julie McKinney, Kelly A. Reynolds, Charles P. Gerba

**Affiliations:** 1 Department of Environmental Science, University of Arizona, Tucson, AZ USA; 2 EPSRC Centre for Doctoral Training in Fluid Dynamics, University of Leeds, Leeds, UK; 3 Department of Community, Environment, & Policy, Mel and Enid Zuckerman College of Public Health, University of Arizona, Tucson, AZ USA; 4 Global Research & Development for Lysol and Dettol, Reckitt Benckiser LLC, Montvale, NJ USA

**Keywords:** QMRA, Virus, Fomites, Hygiene, Public health

## Abstract

Restroom use has been implicated in a number of viral outbreaks. In this study, we apply quantitative microbial risk assessment to quantify the risk of viral transmission by contaminated restroom fomites. We estimate risk from high-touch fomite surfaces (entrance/exit door, toilet seat) for three viruses of interest (SARS-CoV-2, adenovirus, norovirus) through eight exposure scenarios involving differing user behaviors, and the use of hand sanitizer following each scenario. We assessed the impacts of several sequences of fomite contacts in the restroom, reflecting the variability of human behavior, on infection risks for these viruses. Touching of the toilet seat was assumed to model adjustment of the seat (open vs. closed), a common touch point in single-user restrooms (home, small business, hospital). A Monte Carlo simulation was conducted for each exposure scenario (10,000 simulations each). Norovirus resulted in the highest probability of infection for all exposure scenarios with fomite surfaces. Post-restroom automatic-dispensing hand sanitizer use reduced the probability of infection for each virus by up to 99.75%. Handwashing within the restroom, an important risk-reduction intervention, was not found to be as effective as use of a non-touch hand sanitizer dispenser for reducing risk to near or below 1/1,000,000, a commonly used risk threshold for comparison.

**Supplementary Information:**

The online version contains supplementary material available at 10.1007/s12560-023-09580-1.

## Introduction

**Fig. 1:**
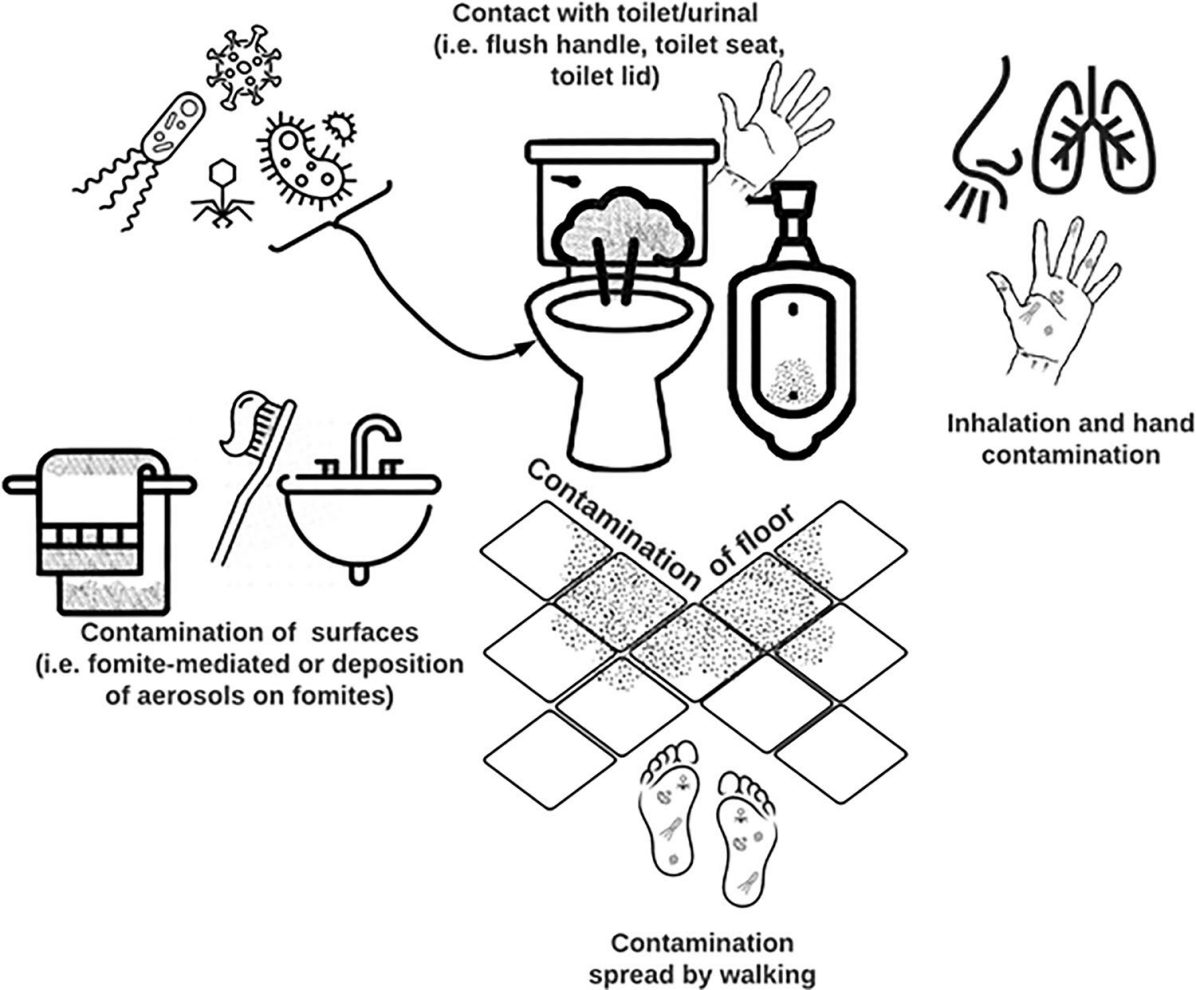
Possible routes of exposure to pathogens, including viruses, in the restroom environment

Restrooms have been implicated as a source of hepatitis A, norovirus, and SARS-CoV-2 outbreaks, and contamination of restroom fomites has been documented in several studies (Abney et al., 2021). Multiple routes of exposure during restroom use can occur, such as accumulation of pathogens on the body and clothes of the user through aerosolization during toilet flushing, direct inhalation of aerosols, or indirect transmission following deposition of aerosolized pathogens on various surfaces throughout the restroom (from use of face towels, contaminated soap bars, or other high-touch surfaces, such as toilet lid, flush handle, faucets, door handles, etc.) (Fig. 1).

Quantitative microbial risk assessment (QMRA) is an approach that is used to assess the risks of infectious agent transmission by water, food, air, and inanimate objects (i.e., fomites) (Haas et al., 2014). This mathematical modeling approach utilizes environmental contaminant data and model annual exposures to determine public health risks. QMRA has been used to develop guidelines for setting standards (i.e., 1:10,000 annual risk) for microbial risk of infection for drinking water by the United States Environmental Protection Agency and regulatory agencies of several countries (Haas et al., 2014). Fomite-mediated transmission has shown to be a sufficient pathway for respiratory and enteric viruses, including rhinovirus, influenza virus, and norovirus, in highly trafficked public venues such as restrooms (Kraay et al., 2018). Due to the lack of guidelines for fomite-mediated risk, other thresholds rooted in the EPA drinking water guidelines (Environmental Protection Agency, 2011) have been developed for this purpose (1:1,000,000 (0.0001%) single event) (Ryan et al., 2014). The quantification of pathogen concentration, transmission, and risk of infection from the use of a typical North American toilet has not been explored for many types of pathogens. The literature that is available for detection of pathogens in restroom environments suggests that this environment represents a likely exposure pathway for certain pathogens (Abney et al., 2021).

Adenovirus is a non-enveloped double-stranded DNA virus with two serotypes (40 and 41) that cause gastroenteritis and are known for their prolonged stability (seven days to three months) in the environment. Carducci et al. (2016) studied the risk of adenovirus infection from aerosols in different occupational settings, including wastewater treatment plants, solid waste landfills, and toilets in healthcare and office buildings. Virological monitoring demonstrated the presence of adenovirus in air samples taken from each of these settings. The results of a QMRA in that study (Carducci et al., 2016) showed that the risks of infection from airborne transmission were the greatest from the aerosols present in public restrooms. They found that the concentration of adenovirus in office building restroom air averaged ∼ 10^5^ genome copies/m^3^, a concentration similar to that recovered from four hospital patient rooms (∼ 10^8^ genome copies/m^3^). It is important to note that fomites can become contaminated through the deposition of aerosols generated during coughing or toilet flushing, and through contaminated hand contact with inanimate objects (Sassi et al., 2018).

Due to the COVID-19 pandemic and uncertainties regarding the potential contribution of fecally-mediated exposures to the transmission of disease, exploration of community transmission of SARS-CoV-2 is necessary. Amoah et al. (2021) estimated the probability of infection of SARS-CoV-2 resulting from touching various surfaces in public restrooms. These authors used qPCR to quantify the numbers of genomic copies of the virus on surfaces. They calculated that the highest annual probability of infection for one-time contact with the various surfaces in the restroom (1.76 × 10^–4^ to 3.79 × 10^–5^ infections per year) was associated with one-time touch with the toilet seat (1.76 × 10^–4^). Risk increased for the various surfaces (4.33 × 10^–4^ to 9.69 × 10^–5^) for three contacts in a day. Infection risks for a one-time exposure may be considered acceptable if below 1 × 10^–6^ (Signor & Ashbolt, 2009).

To our knowledge, there have been five documented instances in which viable SARS-CoV-2 has been isolated from fecal samples (Dergham et al., 2021; Wang et al., 2020; Xiao et al., 2020; Zhang et al., 2020a; Zhou et al., 2020). Dergham et al. (2021) attempted to isolate virus from 106 stool samples from 46 patients testing positive for SARS-CoV-2, with infectious virus being isolated from only 2/106 (1.9%) of the stool samples. The authors reported that the virus was not stable in feces for more than 2 to 6 h. It should be noted that there are difficulties associated with isolation of SARS-CoV-2 from fecal samples using cell culture infectivity assays, making determination of infection risk via the fecal–oral transmission route difficult to assess. Cell culture approaches for assessing the infectivity of wild-type viruses isolated from human specimens and the environment are typically less sensitive than those for laboratory-adapted viruses, because the former have not been adapted to cell culture. For this reason, the true numbers of infectious viruses able to be isolated from field samples are likely to be underestimated (Ward et al., 1984; Zhang et al., 2020b). Although there is insufficient evidence to indicate that the fecal–oral route plays a major role in COVID-19 transmission, it is possible that this transmission pathway contributes to the overall burden of disease.

Norovirus is a non-enveloped positive-sense RNA virus with two genotypes (GI and GII) that are infectious for humans. Both serotypes are highly contagious (10–100 viral particles can lead to an infection) and these serotypes represent the most common cause of acute viral gastroenteritis in humans. Morter et al. (2011) conducted a study in a hospital setting and found norovirus genome copies on 31.4% of swabs for surfaces, including surfaces related to hand hygiene (46.2% of swabs for soap dispensers, 42.9% for alcohol dispensers) and 12.9% of swabs for door handles. The risk of infection from a single fomite in the restroom can be relatively high for norovirus and is affected by the shedding of virus in the diarrhea or vomit of symptomatic patients (Overbey et al., 2021). Norovirus has been found to spread widely to other surfaces within the confined spaces within a houseboat, causing outbreaks (Jones et al., 2007). Barker et al. (2004) showed that norovirus from fecal material can spread via fingers in a sequential manner to up to seven clean surfaces.

The restroom can serve as a reservoir for these pathogens, and contamination may spread well outside of the restroom environment if effective interventions are not observed. Fecal–oral spread of bacteria and viruses, including poliovirus, can occur from restroom surfaces and lead to transfer to objects in the living room and kitchen surfaces within the home (Curtis et al., 2003).

As mentioned above, contamination of the floor in the restroom poses risk of spreading pathogens to other indoor areas following restroom use in offices, hospitals, or homes. Shoes are a potential mode of contamination spread through daily movement and donning and doffing. In a study in a household setting, forty percent of shoe soles were found to have *Clostridium difficile* present on them (Alam et al., 2014). Individuals infected with certain viruses have been shown to contribute to the spread of viruses through contaminating high-touch fomites (Zhang et al., 2020b). Li et al. (2020) showed that toilets cause 40%-60% upward movement (as high as 106.5 cm from the ground) on air plumes of aerosolized particles from the toilet bowl. Aerosolization of viruses from toilets can also influence fomite transmission by depositing virus on the toilet seat or other high-touch surfaces (Goforth et al., 2023; Johnson et al., 2013b). Hand contact with these surfaces (toilet, door handles, faucets) can further spread virus to fomites outside of the restroom.

To our knowledge, there have been no QMRA studies published on the impact of sequences of fomite contacts in a single-user shared restroom environment (home, small business, hospital), specifically, and considering the variability of human behaviors in the restroom on infection risks. From a modeling perspective, there are a paucity of quantitative data on pathogen presence and concentration within restrooms. Although many studies have assessed the presence of microbial pathogens, the reported values have been quasi-quantitative (presence/absence data), making it difficult to relate microbial presence to probability of a health outcome, which requires quantitative data. Changes in human behavior during the COVID-19 pandemic (including, but not limited to social distancing, mask wearing, and hand hygiene) have been shown to reduce not only the spread of respiratory viruses, but of enteric viruses as well (Ijaz et al., 2022). Viral co-infection with rhinovirus and influenza virus has been reported, as well as marked reductions in respiratory viruses, including parainfluenza virus, influenza virus, human coronavirus, and human metapneumovirus, and enteric pathogens (Ahn et al., 2021; Ijaz et al., 2022; Kim et al., 2021a). In Korea, the spread of norovirus and adenovirus was found to be reduced by 40.2% and 13.4%, respectively, during 2 years of heightened COVID-19 personal hygiene and social distancing practices (Kim et al., 2021a, 2021b). However, in the case study by Kim et al. (2021b), enteric pathogen spread increased from 19.4% to 38.3% during level 1 (i.e., the least stringent) social distancing practices in South Korea, demonstrating the impact of fomite transmission despite certain interventions having been implemented, including primarily social distancing and community crowding restrictions, but no stay-at-home orders or business/school closures. These studies emphasize the importance of understanding how human behaviors relate to pathogen transmission risks.

The objective of this study was to assess the probability of infection due to the transmission of three viral pathogens (adenovirus, SARS-CoV-2, and norovirus) with respect to high-touch surfaces (door handles and toilet seats) within the restroom environment. The QMRA was conducted following the compilation of the quantitative data on concentration of viruses within the restroom available in the current literature (Supplementary Information).

## Materials and Methods

**Fig. 2:**
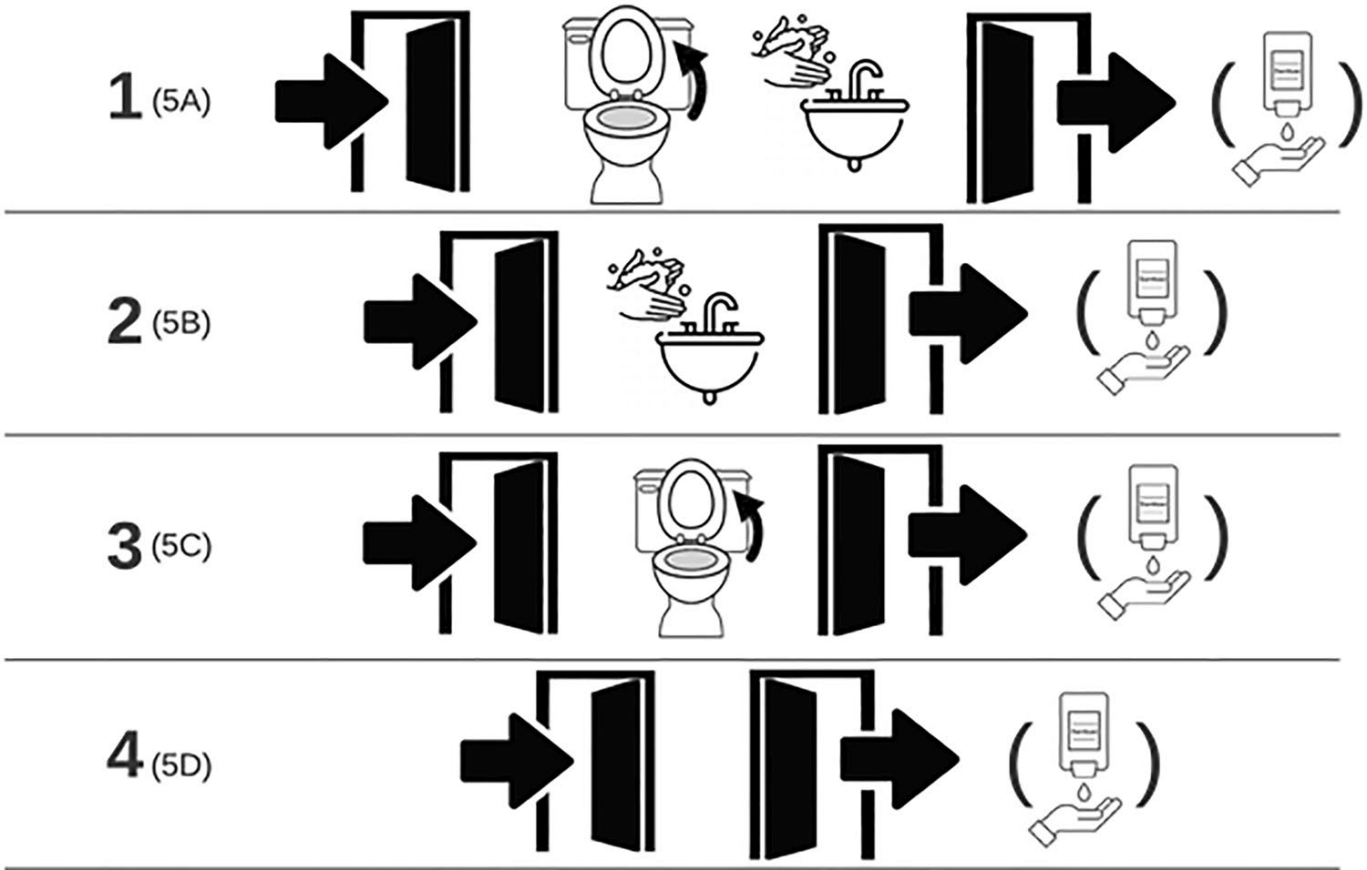
Behavior scenarios evaluated, including added hand sanitization after exiting the restroom

A QMRA modeling approach was used to estimate the probability of acquiring infection with SARS-CoV-2, norovirus, and adenovirus following exposure to published burdens of the viruses on contaminated restroom surfaces (Table 1) using a Monte Carlo simulation (10,000 iterations). Estimated risks were compared to 1:10,000, a commonly used threshold for comparisons of fomite QMRA outputs. However, it should be noted that this risk threshold originated as an annual probability of infection from drinking water (Environmental Protection Agency, 2011). This threshold has been used to determine the need for public health interventions, where risks have been deemed acceptable if below a 1 in 10,000 probability (0.01%) (Abney et al., 2022; Rose & Gerba, 1991; Wilson et al., 2020a). The model scenarios assume a single-user residential or office restroom. Five behavior scenarios were considered (Fig. 2).

**Table 1:** QMRA parameters used to determine probability of infection from fomites contaminated with infectious virus

Parameter	Distribution	References	Notes
Norovirus concentration on the toilet seat (genome copies/cm^2^ in an area contacted by the full hand contact surface area) (*SCa*)	Triangular (lower = 23412.75, mode = 31217, upper = 39021.25)	(Park et al., 2015)	Mode provided by Park et al. (2015) Due to lack of information regarding variance, bounds were assumed based on a variance of 25% for the lower and upper values
Adenovirus concentration on the toilet seat (genome copies/cm^2^ in an area contacted by the full hand contact surface area) (*SCa*)	Left-truncated normal (mean = 222, *SD* = 44, lower = 0)	(Verani et al., 2014)	Mean and SD for the concentration of virus on the toilet seat, left side truncated to eliminate probability of negative values
SARS-CoV-2 burden on the toilet seat (genome copies/cm^2^ in an area contacted by the full hand contact surface area) (*SCa*)	Left-truncated normal (mean = 132.7, *SD* = 252.7, lower = 0)	(Amoah et al., 2021)	Mean and SD for the concentration of virus on the toilet seat, left side truncated to eliminate probability of negative values
Norovirus burden on the entrance/exit door (genome copies/cm^2^ in an area contacted by the full hand contact surface area) (*SCa*)	Triangular (lower = 2006.25, mode = 2675, upper = 3343.75)	(Park et al., 2015)	Mode of door handle virus burden provided by Park et al. (2015), assumed to be similar for the entrance/exit door of a single-user restroom. Due to lack of information regarding variance, bounds were assumed based on a variance of 25% for the lower and upper values
Adenovirus burden on the entrance/exit door (genome copies/cm^2^ in an area contacted by the full hand contact surface area) (*SCa*)	Left-truncated normal (mean = 270, *SD* = 40, lower = 0)	(Verani et al., 2014)	Mean and SD for the burden of virus on the internal door handle (exit door handle), assumed to be equivalent to the external latch (entrance door handle) left side-truncated to eliminate probability of negative values
SARS-CoV-2 burden on the entrance/exit door (genome copies/cm^2^ in an area contacted by the full hand contact surface area) (*SCa*)	Left-truncated normal (mean = 51.5, *SD* = 42.9, lower (left) = 0)	(Amoah et al., 2021)	Mean and SD for the burden of virus on the internal door latch (exit door handle), assumed to be equivalent to the external latch (entrance door handle) left side-truncated to eliminate probability of negative values
Number of genome copies of infectious particles (*SCa*)	Uniform (min = 100, max = 1000)	(Julian et al., 2009)	Minimum and maximum assumption of fraction of infectious viral particles (1/100–1/1000)
Toilet seat surface-to-hand transfer efficiency (fraction) (*TEs*2ℎ)	Truncated Normal (mean = 0.3445, *SD* = 0.138, min = 0.186, max = 0.55)	(Lopez et al., 2013)	Mean and SD transfer efficiencies reported for low and high relative humidity conditions for phage MS2 for laminate surfaces (closest surface composition to a toilet seat)
Doorknob surface-to-hand transfer efficiency (fraction) (*TEs*2ℎ)	Truncated Normal (mean = 0.2215, *SD* = 0.1245, min = 0.1045, max = 0.433)	(Lopez et al., 2013)	Mean and SD transfer efficiencies reported for low and high relative humidity conditions for phage MS2 for stainless steel (closest surface composition to a doorknob)
HCoV 229E toilet seat surface-to-hand transfer efficiency (%) (*TEs*2ℎ)	Truncated Normal (mean = 0.4907, *SD* = 0.1667, lower (left) = 0, upper (right) = 1)	(Gerba et al., 2023)	Mean and SD transfer efficiencies reported for HCoV 229E on glazed porcelain
HCoV 229E doorknob surface-to-hand transfer efficiency (%) (*TEs*2ℎ)	Truncated Normal (mean = 0.0046, *SD* = 0.0057, lower (left) = 0, upper (right) = 1)	(Gerba et al., 2023)	Mean and SD transfer efficiencies reported for HCoV 229E on stainless steel
Hand-to-facial mucous membrane transfer efficiency (%) (*TE*ℎ2*f*)	Normal (mean = 0.3390, *SD* = 0.15) Left- and right-truncated at 0 and 1, respectively	(Rusin et al., 2002)	The mean and SD for transfer efficiency originates from Rusin et al. (2002) Updated variances have been provided by Abney et al. (2022)
Fraction of total hand surface area used for hand to-facial mucous membrane contact (*Af*)	Uniform (min = 0.008, 0.012)	(AuYeung et al., 2008)	Assumed a single fingertip would be used for this contact. Divided the minimum and maximum fractions of the left and right hand for adults for partial front finger configurations by five
Fraction of total hand surface area used for door contact (*Af*)	Uniform (min = 0.10, 0.017)	(AuYeung et al., 2008)	Assumed closed grip would be used for contact with the door handle to open or close
Fraction of total hand surface area used for toilet seat contact (*Af*)	Uniform (min = 0.01, 0.04)	(AuYeung et al., 2008)	Assumed a pinch grip would be used to adjust the toilet seat
Total hand surface area (cm^2^) (*A*ℎ)	Uniform (min = 445, max = 535)	(Beamer et al., 2015) (Environmental Protection Agency, 2011)	Table 7–2 mean surface area of hands for adult females and males 21 + estimated from analysis of NHANES data, where total hand surface area here is for a single hand (total for both hands divided by 2)
Log_10_ reductions for handwashing (*HW*)	Uniform (min = 0.58, max = 1.58)	(Liu et al., 2010)	Minimum and maximum log_10_ reductions reported for handwashing with soap
Log_10_ reductions for hand sanitizer (*HS*)	Uniform (min = 2, max = 4)	(Kampf et al., 2020)	Minimum and maximum log_10_ reductions reported for use of hand sanitizer

*HCoV* human coronavirus, *min* minimum, *max* maximum, *SD* standard deviation

Scenario 1: A contact is made with the entrance door, then the toilet seat (to lift it or lower it) followed by a handwashing intervention and then contact with the exit door. This models the use of the restroom, assuming adjustment for preference of use (i.e., males adjusting the toilet seat with lifting and females adjusting the toilet seat down, if already up) prior to use. This also captures a hand hygiene intervention prior to exiting the restroom.

Scenario 2: A contact is made with the entrance door followed by a handwashing intervention and then contact with the exit door. This models the use of the restroom to, for instance, wash hands before eating or after doing laundry.

Scenario 3: A contact is made with the entrance door, then with the toilet seat (to lift it or lower it) assuming adjustment for gender preference, followed by a contact with the exit door. This models use of the restroom with gender-differences in the preference of the toilet seat, while also capturing the lack of hand hygiene prior to exiting the restroom.

Scenario 4: A contact is made with the entrance door, and then with the exit door. This models the use of the restroom for other activities not including the use of the toilet or hand hygiene (for instance, use of mirror, picking up an item).

Scenario 5: Scenarios 5A-5D are the same as Scenarios 1 through 4, respectively, in each case with added use of non-touch hand sanitizer dispenser after exiting the restroom.

For each scenario, probability of infection from a single restroom visit and probability of infection from three restroom visits in one day were quantified. For three restroom visits in one day, the estimation is based on surface virus burdens from the literature. Model parameters are detailed in Table 1. We assume virus burdens on surfaces are not influenced by multiple uses over time, since we are focusing on single-use scenarios (Fig. 2). Therefore, we do not assume cumulative increase in surface virus burdens over multiple days.

We assumed that the starting hand virus burdens were zero at the beginning of each restroom visit, as we lacked the data to properly estimate surface contamination outside of the restroom prior to each restroom visit. It is likely that any virus present on hands would be lost due to hand hygiene interventions outside of the restroom and transfer of virus to other contaminated surfaces.

### Virus Concentration Changes Due to Contacts with Fomites

All surface burdens of virus (in units of genome copies (gc) per cm^2^) (Table 1) were adjusted to represent infectious particles per cm^2^. A ratio of infectious particles to genome copies was randomly sampled from a uniform distribution (minimum = 10, maximum = 100) (Pitol et al., 2018). The virus burden on the hand after the first contact with the door handle was calculated, taking into account the fraction of the total hand surface area used (*Af*), the surface burden (*Sca)*, and the transfer efficiency (*TEs2h*) (Eq. 1).

1 $${\text{Hand Virus Concentration}}\left( {HC} \right)~ = ~\left[ {SC_{{\text{a}}} \times ~\left( {Af} \right) \times TEs2h} \right]$$

The fraction of the hand surface used for the contact ($Af$) was informed by the minimum and maximum fractions of the left and right hand for adults for a pinch grip (to represent the most likely grip to raise/lower a toilet seat) or closed hand grip (to represent grabbing a doorknob) (AuYeung et al., 2008) (Table 1).

When no hand hygiene was simulated (scenarios three and four), virus burden on the hand was calculated for each sequential contact without any reductions due to hand hygiene. An example of how this was done for Scenario 3 is given below.

2 $$T_{{1 \to 2}} = \left( {HC} \right) - \left[ {\left( {TEs2h \times Af} \right)\left( {HC - SC_{{\text{a}}} } \right)} \right]$$

3 $${T}_{2\to 3} ={(T}_{1\to 2})-\left(TEs2h\times Af\right){(T}_{1\to 2}-{SC}_{a})$$

The term *HC* is the amount of virus found on the hand after one surface touch with the entrance door (Eq. 2). The virus burden on the hand after a second contact (toilet seat) was then calculated using Eq. 2, where virus transfer occurs in a bi-directional manner (i.e., both addition of virus to and removal of virus from the hand). The new virus burden on the hand was calculated as a function of transfer efficiency ($TEs2h$), fraction of the hand used for the contact ($Af$), and the virus burden on the toilet surface ${(T}_{1\to 2}$). Transfer efficiency for adenovirus and norovirus was informed by phage MS2 transfer experiments using coupons similar in composition to that of the toilet seat (laminate) and door handle (steel) (Gerba et al., 2023; Lopez et al., 2013; Rusin et al., 2002). MS2, a non-enveloped bacteriophage often used as a surrogate for norovirus (for which robust infectivity assays do not exist), was assumed to be a surrogate also for adenovirus in this model. There is a lack of literature regarding the transfer of adenovirus from fomites to fingers without absorption (Dawson et al., 2005; Pitol et al., 2018). Transfer efficiency for SARS-CoV-2 was informed by recent transfer efficiency experiments performed with HCoV 229E (human coronavirus 229E) from a toilet seat surface and steel plate (doorknob) to fingerpads (Gerba et al., 2023). Human coronavirus 229E has proven to be a relevant surrogate for SARS-CoV-2 through survivability and inactivation experiments and it displays phylogenetic similarity (Butot et al., 2021; Forster et al., 2020; Kampf et al., 2020).

The burden of virus on the finger after touching the second surface (toilet seat) (${T}_{1\to 2})$ then was used to calculate the viral load on the finger after touching the third surface (exit door handle) (${T}_{2\to 3})$ (Eq. 3).

### Handwashing

The burden of virus on the hand following contact with the entrance door or toilet seat was used to calculate reduction due to hand washing (Eq. 4).

4 $$HC\left( {T_{{X \to HW}} } \right) = \left[ {\left( {HC\left( {T_{{1 \to 2}} } \right)} \right) \times \left( {\frac{1}{{10^{{hw}} }}} \right)} \right]$$

### Hand Sanitizer

When hand sanitizer use was modeled, the final virus burden on the hand after contact with the exit door handle was reduced by an expected log_10_ reduction (Eq. 5).

5 $$HC\left( {T_{{3 \to HS}} } \right)~ = \left( {HC\left( {T_{{2 \to 3}} } \right)} \right)\left( {\frac{1}{{10^{{hs}} }}} \right)$$

### Viral Dose

Doses for scenarios with and without hand sanitizer use were calculated (Eq. 6**)** by estimating how much of the viral load on a person’s hand at the end of fomite contact sequences might transfer to the face during a hand-to-face contact.

6 $$T_{{FC \to D}} = HC \times TEh2f \times Af \times Ah$$

Dose (${T}_{FC\to D}$) was calculated with the final burden of virus on the hand ($HC({T}_{X\to HW})$ or $({HC(T}_{3\to HS}$)), irrespective of the scenario under evaluation, along with parameters including the total surface area of the hand ($Ah$), the fraction of the hand used for contact with a mucosal membrane (mouth, eyes, nose) ($Af$), and the transfer efficiency from the hand to facial mucosal membrane (*TEh2f*) contact. The fraction of the hand used for the contact ($Af$) was informed by the minimum and maximum fractions of the left and right hand for adults for partial front finger configurations divided by five, in order to assume contact of a single fingertip (AuYeung et al., 2008). Transfer efficiency from hand-to-face was informed by Rusin et al. (Rusin et al., 2002), which represents, to our knowledge, the only microbial transfer data available for hand-to-lip contact.

### Dose Response

Risk of infection was calculated from doses of all five multiple surface touch scenarios, for both a single restroom visit and for three restroom visits (representing daily risk) (Table 2).

**Table 2:** Viral dose–response parameters

Pathogen	Dose response curve	Dose response parameter	Study
Norovirus	Beta-Poisson	*α* = 0.1	(Teunis et al., 2008) (mixed aggregation and dispersion) (Overbey et al., 2021)
		*ẞ* = 32.3	
Adenovirus	Exponential	*k* = 0.607	(De Albuquerque et al., 2006) interpreted by Haas et al. (Haas et al., 2014)
SARS-CoV-2	Exponential	*k* = Triangular (0.0011, 0.00256, 0.0068)	(DeDiego et al., 2008; Haas et al., 2014; Marin-Gomez et al., 2021; Pitol & Julian, 2021)

*α, ẞ, *constants for Beta-Poisson equation

*k, *constant from exponential equation

Beta-Poisson:

7 $$P\left( {{\text{response}}} \right) = 1 - 1[1 + \left( {\frac{{{\text{dose}}}}{\beta }} \right)]^{{ - \alpha }}$$

Exponential:

8 $$P~\left( {{\text{response}}} \right) = ~1 - \exp \left( { - k~ \times ~{\text{dose}}} \right)$$

The approximate Beta-Poisson curve (Eq. 7) was used for norovirus, with the assumption of both virus aggregation and disaggregation (Teunis et al., 2008). Van Abel et al. (2017) found, through the analysis of multiple norovirus dose–response models, that although many published QMRAs assume aggregation, the best practice is to select the dose–response curve based on the assumption of anticipated virus aggregation behavior within risk models, as this can greatly impact predicted risk. We assume that, because the virus is suspended in a fecal matrix, there will be mixed aggregation due to the complex proteinaceous composition of the human excreta (Itoh et al., 2000). Adenovirus and SARS-CoV-2 were modeled with an exponential dose–response curve, with parameters informed by the QMRA Wiki (“Completed Dose Response Models | QMRA” 2021) (Eq. 8). Out of two available dose–response curves on QMRA Wiki, the adenovirus dose–response curve from Couch et al. (1966) was chosen, as it more closely resembled that for fomite-mediated transmission. The SARS-CoV-2 dose–response model chosen has been used in prior literature to model community transmission via fomites (Pitol & Julian, 2021).

Using the summation of these equations appropriate to the five different scenarios and three different viruses, we were able to associate probability of infection mathematically modelled and statistically analyzed using R version 4.0.2 (RStudio Team, 2022). We report risk in percent risk, as we believe it is easier for our wider audience (the general public) to understand risk expressed in this manner and the changes in risk due to different environmental influences (i.e., number of items touched, etc.). We also think it is useful or the scientific community and public-at-large to remove the jargon around public health risk estimations. Note that the U.S. EPA public health guideline (Environmental Protection Agency, 2011) for annual risk is 1:10,000 and, according to the sample calculation noted in Ryan et al. (2014), the daily risk is 1:1,000,000. The daily risk more closely approximates the risk associated with a single restroom visit. This U.S. EPA-based public health guideline is appropriate for fomite transmission risk as calculated in Eq. 9. The guideline recommends that a 0.0001% risk or higher be considered of concern.

9 $$P~\left( {\% ~{\text{risk}}} \right) = \left[ {P~\left( {{\text{response}}} \right)] \times 100} \right]$$

### Sensitivity Analysis

The monotonic relationships between input variables (viral burden on each high-touch surface, transfer efficiency, fraction of total hand surface area used in fomite contact, and efficacy of viral reduction interventions), and infection probability were quantified using Spearman correlation coefficients. A Spearman correlation coefficient of greater absolute magnitude indicates a stronger relationship. This method has been used in other QMRA sensitivity analyses (Canales et al., 2019; Pitol & Julian, 2021; Wilson et al., 2020b).

## Results

Estimated probability of infection associated with adjusting a toilet seat varied widely among the three viruses of interest evaluated (Table 3). Percent probability of infection for each virus represents risk associated with touching fomites in the restroom with one finger, with or without hand hygiene (depending upon the scenario), followed by a hand-to-mucosal membrane contact. The highest calculated mean probability of infection was for norovirus across all scenarios. The highest risk scenario for all viruses was touching all three high-touch points (entrance door, toilet seat, and exit door) (Scenario 3).

**Table 3:** Risk of infection (% probability) (mean ± *SD*) for a multiple touch event of high-touch surfaces within a restroom

Exposure scenario	One daily restroom use (%)			Three times daily restroom use (%)		
	Adenovirus	Norovirus	SARS-CoV-2	Adenovirus	Norovirus	SARS-CoV-2
Scenario 1 (Enter > Adjust Toilet > Wash > Exit)	0.39 ± 0.39	1.58 ± 1.27	2.63 × 10^–4^ ± 2.79 × 10^–4^	1.17 ± 1.15	3.88 ± 2.60	7.89 × 10^–4^ ± 8.38 × 10^–4^
Scenario 2 (Enter > Wash > Exit)	0.38 ± 0.38	1.11 ± 0.98	6.27 × 10^–5^ ± 8.50 × 10^–5^	1.14 ± 1.13	2.85 ± 2.13	1.88 × 10^–4^ ± 2.55 × 10^–4^
No Hand Wash Scenario 3 (Enter > Adjust Toilet > Exit)	0.78 ± 0.75	1.49 ± 1.21	2.56 × 10^–3^ ± 3.06 × 10^–5^	2.31 ± 2.18	3.71 ± 2.51	7.68 × 10^–3^ ± 9.18 × 10^–3^
Scenario 4 (Enter > Exit)	0.68 ± 0.68	1.19 ± 1.04	1.16 × 10^–4^ ± 1.61 × 10^–4^	2.02 ± 1.97	3.04 ± 2.24	3.48 × 10^–4^ ± 4.83 × 10^–3^

Probabilities of infection based on U.S. EPA public health guideline exceeding 0.0001% (1:1,000,000) are of concern (Ryan et al., 2014)

Comparison of one-time vs. three-time restroom use within a single day was performed to highlight the risk of using the restroom (one time) and a minimum daily risk (three-time use). Scenario 3 for a three-time use (daily risk) for norovirus resulted in a risk of 3.71%, relative to a risk of 1.49% for a one-time use. The risks associated with three-time use and one-time use of the restroom in exposure scenarios did not vary by more than 1% for adenovirus and less than 3% for norovirus and SARS-CoV-2. Probability of infection for SARS-CoV-2, even for three-time restroom use, was low, with risks being slightly above 1 × 10^–4^% (actual probability of infection is 1 × 10^–6^) for all scenarios except Scenario 3 daily risk (7.68 × 10^–3^%). The largest difference in percent risk between one-time use and three-time use for adenovirus (1.5% difference) and SARS-CoV-2 (0.005% difference) was in Scenario 3. However, norovirus exhibited the largest difference between one- and three-time use for both Scenario 1 (2.3% difference) and Scenario 3 (2.2% difference).

The inclusion of a hand washing intervention prior to exiting the restroom did not greatly decrease risk for SARS-CoV-2 and norovirus (percent probability of infection decrease of 5.33 × 10^–5^% ± 7.6 × 10^–5^% and 0.08% ± 0.06%, respectively, for Scenario 2 vs. Scenario 4).

Scenarios 1–4 were also investigated with post-restroom exit use of hand sanitizer (Fig. 2). Touching the toilet seat without hand washing was identified as the highest risk for all three viruses, so we expanded the QMRA to include hand sanitization after exiting the restroom (Table 4).

**Table 4:** Risk of infection (% probability) for a multiple-touch event of high-touch surfaces within a restroom with hand sanitizer use after exiting the restroom

Exposure scenario	One daily restroom use (%)			Three times daily restroom use (%)		
	Adenovirus	Norovirus	SARS-CoV-2	Adenovirus	Norovirus	SARS-CoV-2
Scenario 5A (Enter > Adjust > Wash > Exit > Sanitizer)	8.34 × 10^–4^ ± 1.59 × 10^–3^	3.90 × 10^–3^ ± 7.53 × 10^–3^	5.59 × 10^–7^ ± 1.13 × 10^–7^	2.50 × 10^–3^ ± 4.77 × 10^–3^	1.2 × 10^–2^ ± 2.25 × 10^–2^	1.68 × 10^–6^ ± 3.38 × 10^–6^
Scenario 5B (Enter > Wash > Exit > Sanitizer)	8.12 × 10^–4^ ± 1.55 × 10^–3^	2.62 × 10^–3^ ± 5.01 × 10^–3^	1.33 × 10^–7^ ± 3.32 × 10^–7^	2.44 × 10^–3^ ± 4.65 × 10^–3^	7.85 × 10^–3^ ± 1.5 × 10^–2^	3.98 × 10^–7^ ± 9.97 × 10^–7^
No Hand Wash Scenario 5C ***(***Enter > Adjust > Exit > Sanitizer)	1.66 × 10^–3^ ± 3.08 × 10^–3^	3.64 × 10^–3^ ± 6.77 × 10^–5^	5.38 × 10^–6^ ± 1.11 × 10^–5^	4.97 × 10^–3^ ± 9.25 × 10^–3^	1.09 × 10^–2^ ± 2.02 × 10^–2^	1.61 × 10^–5^ ± 3.32 × 10^–5^
Scenario 5D: ***(***Enter > Exit > Sanitizer)	1.45 × 10^–3^ ± 2.74 × 10^–3^	2.84 × 10^–3^ ± 5.43 × 10^–3^	2.45 × 10^–7^ ± 6.25 × 10^–7^	4.35 × 10^–3^ ± 8.23 × 10^–3^	8.52 × 10^–3^ ± 1.62 × 10^–2^	7.35 × 10^–7^ ± 1.87 × 10^–6^

These scenarios demonstrated that the use of hand sanitizer after exiting the restroom was the most effective intervention (superior to hand washing within the restroom) for reducing probability of infections for each of the three viruses of interest, with a maximum percent decrease of 99.75% (norovirus, Scenario 3 vs. Scenario 5C). SARS-CoV-2 risk from three-time daily restroom use for Scenario 3 at a probability of infection of 2.56 × 10^–3^% was reduced to a risk of infection of less than 1 × 10^–4^% through addition of use of hand sanitizer after exiting the restroom (probability 5.38 × 10^–6^%) (Scenario 5C).

## Discussion

This QMRA modeling exercise demonstrates that the greatest risk scenario across each of the viruses evaluated involves contact with the entrance door, toilet seat, and exit door without a hand washing intervention (Scenario 3). Although this model shows probability of infection across multiple viruses presumed to involve the fomite route of transmission, it provides an insight into the varying degrees of infection risk for different viruses in the restroom due to their infectivity/environmental concentrations and demonstrates that hand hygiene can be effective, if used in the proper sequence. High prevalence of pathogen contamination of the exit door and toilet seats for restrooms has been previously noted (Amoah et al., 2021; Park et al., 2015; Verani et al., 2014). However, how risk varies across the viruses was evaluated here. Hand washing played the greatest role in risk reduction in the case of adenovirus but played a smaller role in reduction of risk in the cases of SARS-CoV-2 and norovirus, compared with hand sanitizer use. We suggest in this model the addition of a new risk reduction intervention sequence: a hand sanitizer dispenser outside of the restroom that automatically dispenses sanitizing agent (i.e., touch-free, to avoid introduction of an additional touch point). We stress the automation of the dispenser, as Mortar et al*.* (2011) found that nearly half (42.9%) of mechanical dispensers tested were contaminated with norovirus, and 50% of those were still contaminated after hospital-grade cleaning. The addition of hand sanitizer use following each scenario (Scenarios 5A-5D) greatly reduced probability of infection in this QMRA, with up to 99.8% reduction in the case of Scenario 5D. Touching the toilet seat, as opposed to not touching the toilet seat, for one-time use (Scenario 3 vs. Scenario 4) differed in risk by 0.1% for adenovirus (0.78% (scenario 3) to 0.68% (scenario 4)), 0.03% for norovirus (1.19% to 1.49%), and 2.4 × 10^–3^% for SARS-CoV-2 (1.16 × 10^–4^% to 2.56 × 10^–3^%).

Previous risk assessments have demonstrated a high probability of infection, exceeding 10^−4^ (0.01%) annual risk of infection, for those exposed to pathogens such as adenovirus while using the restroom (Amoah et al., 2021; Carducci et al., 2016; Dada & Gyawali, 2021; Signor & Ashbolt, 2009). However, only Amoah et al. (2021) estimated risk from fomite-mediated transmission, with the highest risk representing the toilet seat (1.8 × 10^–4^%). The other studies estimated risks of 0.3% or higher through inhalation of aerosolized infectious agents (Carducci et al., 2016; Dada & Gyawali, 2021).

Hand sanitizers have been demonstrated to represent an effective risk intervention for viruses that cause respiratory and gastrointestinal illness, and their associated diseases (Sandora et al., 2005; Tamimi et al., 2015; Wilson et al., 2020b). Across all scenarios and selected transfer efficiency data, the use of hand sanitizer after exiting the restroom greatly reduced the probability of infection for each of the viruses evaluated. We found that even if hands were washed with soap and water after touching the toilet seat, infection risk subsequently increased as a result of interaction with the door upon exiting the restroom. We were unable to provide insight in this model regarding further contamination after exiting the single-user restroom, due to the lack of current literature containing quantitative data, however the use of hand sanitizer upon exit should greatly reduce the possibility of contamination spread to surfaces outside of the restroom.

One limitation of the QMRA model developed here is the lack of sufficient data to estimate risk of an entire restroom event (i.e., actually using the toilet vs. just touching the seat). More data are needed that describe sequences of surfaces touched, the frequency of restroom visits, and the frequency of fomite contacts or hand hygiene interventions between restroom visits. Additionally, data are needed regarding the burdens of pathogens on various surfaces in the restroom environment. An in-depth review of current literature on restroom/toilet hygiene has been published by Abney and coworkers (Abney et al., 2021). Additional to that review, we have compiled data for burdens of virus, bacteria, and protozoa/cysts recovered within the restroom (Supplementary Information) and have found that quantitative data regarding the occurrence of pathogens on high-touch fomites within the restroom, as well as the viability of such pathogens (i.e., infectivity vs. genomic copies), is much needed to advance the knowledge of risk while using the restroom –

While SARS-CoV-2 has been detected in feces, epidemiological data supporting COVID-19 transmission via exposure to fecal matter is lacking (Wang et al., 2020; Xiao et al., 2020; Zhang et al., 2020b; Zhou et al., 2020). Further advancements in methods for isolation of virus from fecal samples are needed (Dergham et al., 2021). Therefore, the possibility of transmission through the fecal–oral route requires further investigation. Additionally, this QMRA model did not account for various human excreta and bodily fluids expected to be generated within restroom environments (i.e., feces, urine, blood, vomit, sputum) and the extent to which these excreta might impact transfer efficiency. Representative matrices used to mimic fomite contamination and human excreta composition have not been widely explored, yet available data have highlighted transfer efficiency differences based on commonly used model excreta matrices (Abney et al., 2022). In addition to effecting transfer efficiency, the excreta may introduce varying concentrations of pathogens, depending upon how pathogens are shed by infected individuals. For example, through modeling, Overbey et al. (2021) demonstrated that exposure to diarrhea in the restroom of symptomatic patients increased probability of infection by four orders of magnitude, relative to exposure to vomit, based on pathogen concentration data. Inclusion of transfer efficiencies that best model different human excreta, as shown by Abney et al. (2022), with the addition of concentration data for viruses in excreta/bodily fluids (i.e., sputum, diarrhea, vomit, saliva) which are not yet available, would increase the specificity for a particular pathogen of QMRA models for identifying risk, not only in the restroom, but for fomite-mediated transmission models in general.

Aerosols (often termed the toilet plume) are produced when flushing a toilet and can pose an additional infection risk in single-user restrooms (the scenarios modeled in this study) or multi-user restrooms. Several studies have investigated such aerosols (Barker & Jones, 2005; Gerba et al., 1975; Jessen, 1955; Johnson et al., 2013a). Microbial tracer studies have shown that droplets in the toilet plume may contain bacteria and viruses, and may remain airborne for an extended period of time, allowing dispersion throughout the restroom (Barker & Jones, 2005; Gerba et al., 1975; Jessen, 1955; Johnson et al., 2013a). Johnson et al. (2013a) found that up to 145,000 droplets can be produced per flush (95% of droplets being < 2 µm and > 99% being < 5 µm in diameter). Under standard atmospheric conditions, droplets less than 100 µm typically evaporate before they settle due to gravity (Morawska, 2006). The evaporated droplet residues (droplet nuclei) may remain airborne for prolonged periods of time, traveling considerable distances due to indoor airflows. Future studies may, therefore, need to consider the risk posed by airborne pathogens derived from the toilet plume by considering inhalation as an exposure route and the mechanisms by which toilet flushing contaminates surrounding fomites.

## Conclusions

Shared restrooms are a potential source in the built environment for the transmission of viral pathogens. Use of these facilities is necessary by all individuals, and a number of surfaces must be touched by individuals using these facilities. Maeda et al. (2015) showed that norovirus infections exhibited a co-infection rate with other pathogens of 25.4% and adenovirus a co-infection rate of 29.4%. Co-infections with SARS-CoV-2 occurred with rhinovirus (5%), influenza A virus (2%), mastadenovirus (1%), and norovirus (1%) in a cohort of 92 patients (Kim et al., 2021a). Oristo et al. (2017) detected norovirus and adenovirus concurrently on participants' hands and surfaces within the restroom. Multiple enteric viruses have been detected in the same restroom samples taken from healthcare settings and food surface operations, showing risk of community spread and outbreak (Maunula et al., 2017; Verani et al., 2014). More research is needed regarding the burden of viruses in the restroom and the spread of contamination of such viruses within and outside the restroom to truly advance our knowledge of the risk and impact that North American toilets might have on public health.

In this study, we used QMRA to confirm that touching of the toilet seat, a previously confirmed high-risk surface, elevates total probability of infection for those visiting the restroom, relative to visits to the restroom that do not involve hand-to-toilet seat contact (using the restroom solely to wash one’s hands, for example). Through the differentiation of behavior sequences in a fomite exposure pathway highlighted in this model, we established that interaction with the exit door (an inevitable contact) increases probability of infection for each of the viruses evaluated. While handwashing is a recommended risk intervention, we demonstrated in our QMRA model that the use of touch-free hand sanitizers as a risk reduction method to be used after exiting the restroom offers additional risk reduction to a risk lower than U.S. EPA public health guideline (1:10,000 or 0.01%).

### Supplementary Information

Below is the link to the electronic supplementary material.

Supplementary file1 (DOCX 28 KB)

## Data Availability

Data used in this study is available from the corresponding author on request.
