# Supplementary material for: Transmission of Viruses from Restroom Use: A Quantitative Microbial Risk Assessment
Source: Food Environ Virol. 2024 Feb 19;16(1):65–78. doi: 10.1007/s12560-023-09580-1 (PMC10963455; doi:10.1007/s12560-023-09580-1)
Supplement: Supplementary file 1 — Supplementary file1 (DOCX 28 KB) [file 12560_2023_9580_MOESM1_ESM.docx]

Transmission of Viruses from Restroom Use: A Quantitative Microbial Risk Assessment

Sarah E. Abney • Ciara A. Higham • Amanda M. Wilson • M. Khalid Ijaz • Julie McKinney • Kelly A. Reynolds • Charles P. Gerba

**Table S1. Spearman correlation results.**

|  |  | Scenario 1 | Scenario 2 | Scenario 3 | Scenario 4 | Scenario 5 |
| --- | --- | --- | --- | --- | --- | --- |
| Adenovirus | TE toilet seat to hand | 0.043817 | -0.00145 | 0.177229 | -0.00078 | 0.082682 |
|  | TE door to hand | 0.326025 | 0.401249 | 0.084736 | 0.363331 | 0.03382 |
|  | TE hand to face | 0.486593 | 0.47534 | 0.515345 | 0.486065 | 0.231922 |
|  | Fraction infectious | -0.67747 | -0.66148 | -0.73068 | -0.68106 | -0.34714 |
|  | Total hand surface area | 0.069959 | 0.066861 | 0.074506 | 0.068764 | 0.035006 |
|  | Fraction of hand used | 0.276994 | 0.277103 | 0.263896 | 0.268344 | 0.117364 |
|  | Hand washing effectiveness | -0.15133 | -0.05926 | ******* | ******* | ******* |
|  | Hand sanitizer effectiveness | ******* | ******* | ******* | ******* | -0.86528 |
|  | Starting conc on toilet seat | 0.677038 | 0.660942 | 0.730481 | 0.680514 | 0.34771 |
|  | Starting conc on entrance door | 0.676876 | 0.66096 | 0.730035 | 0.681005 | 0.346042 |
|  | Starting conc on exit door | 0.678018 | 0.662191 | 0.730303 | 0.68122 | 0.346918 |
| Norovirus | TE toilet seat to hand | 0.037444 | -0.00032 | 0.266739 | -0.00022 | 0.167799 |
|  | **Lopez** TE door to hand | 0.346694 | 0.405517 | 0.145788 | 0.399348 | 0.088126 |
|  | **Rusin** TE hand to face | 0.481008 | 0.469327 | 0.659221 | 0.471658 | 0.411515 |
|  | Fraction infectious | -0.6724 | -0.65622 | -0.48205 | -0.66003 | -0.30278 |
|  | Total hand surface area | 0.068505 | 0.06559 | 0.086798 | 0.065919 | 0.05462 |
|  | Fraction of hand used | 0.278432 | 0.276051 | 0.376788 | 0.274771 | 0.229137 |
|  | Hand washing effectiveness | -0.10453 | -0.0164 | ******* | ******* | ******* |
|  | Hand sanitizer effectiveness | ******* | ******* | ******* | ******* | -0.7489 |
|  | Starting conc on toilet seat | 0.661399 | 0.642246 | 0.483826 | 0.646053 | 0.303818 |
|  | Starting conc on entrance door | 0.662498 | 0.646542 | 0.477255 | 0.65176 | 0.300946 |
|  | Starting conc on exit door | 0.679314 | 0.665448 | 0.476245 | 0.667806 | 0.299606 |
| SARS-CoV-2 | TE toilet seat to hand | -0.00799 | 0.013797 | -0.00557 | 0.014624 | -0.00394 |
|  | TE surface to hand | -0.00106 | -0.00265 | -0.01014 | -0.00323 | -0.01145 |
|  | TE hand to face | 0.384937 | 0.353389 | 0.462556 | 0.352717 | 0.223566 |
|  | Fraction infectious | -0.52597 | -0.49435 | -0.66389 | -0.49598 | -0.34027 |
|  | Total hand surface area | 0.066086 | 0.049187 | 0.071731 | 0.048446 | 0.0378 |
|  | Fraction of hand used | 0.217731 | 0.218807 | 0.27097 | 0.21843 | 0.133667 |
|  | Hand washing effectiveness | -0.58667 | -0.04345 | ******* | ******* | ******* |
|  | Hand sanitizer effectiveness | ******* | ******* | ******* | ******* | -0.84136 |
|  | Starting conc on toilet seat | 0.525967 | 0.494349 | 0.663892 | 0.49598 | 0.340274 |
|  | Starting conc on entrance door | 0.525074 | 0.494048 | 0.663019 | 0.49609 | 0.34005 |
|  | Starting conc on exit door | 0.5256 | 0.494941 | 0.663166 | 0.49616 | 0.339439 |

***** =** variable was not used to calculate risk in scenario

**Table S2. Concentration of Viruses detected in Restrooms.**

| **Virus** | **Location** | **Virus levels** | **Reference** |
| --- | --- | --- | --- |
| Adenovirus | Office restrooms | 124 +/- 42 gc/cm2 | Verani et al 2014 |
|  | Hospital Restrooms | 349 +/- 51 gc/cm2 | Verani et al 2014 |
|  | Office and Hospitals | Door handle 270 +/- 40  flush button 144 +/- 60  toilet seat 222 +/- 44  Toilet cover 312 +/- 63 | Verani et al 2014 |
| Norovirus | Cruise ships with norovirus outbreak | Toilet seat  Cabin A 31,217 gc/700 cm2  Cabin B 986 gc/700 cm2 | Park et al 2015 |
| Crassphage | Cruise ships with norovirus outbreaks | Toilet seats 3.3 ave (1.2 -5.6)/700 cm2?  Toilet door handles ave 2.3 (1.0-3.1) gc/700 cm2? | Park et al 2020 |
| SARS-CoV-2 | Shared toilet - has data before and after cleaning – has QMRA of touching surfaces | Toilet seat ave 130 gc/cm2  Flush handle 70 gc/cm2  Internal latch 50 gc/cm2  Tap handle 30 gc/cm2  Floor | Amoah et al 2020 |
| Rotavirus | Have not yet found quantitative data |  |  |

***** =** variable was not used to calculate risk in scenario

**Table S3. Concentration of bacterial pathogens on toilet surfaces**

| **Risk Parameter** | **Units** | **Value** | **Reference** |
| --- | --- | --- | --- |
| Gram positive on public toilets | cfu/ml | 0.1625×10^5 | Nigatu 2014 |
| Gram Negative on public toilets | cfu/ml | 0.1985×10^5 | Nigatu 2014 |
| Bacteria on toilet entrance door handle (Female) | % | 41.7 | Nworie 2012 |
| Bacteria on toilet knob (Female) | % | 11.5 | Nworie 2012 |
| Bacteria on toilet entrance door handle (male) | % | 36.5 | Nworie 2012 |
| Bacteria on toilet knob (male) | % | 10.3 | Nworie 2012 |
| LIC (Low-income country) Flies in hospital with Shigella | % | 8 | Khalil 2009 |
| LIC (Low-income country) Water in hospital with Shigella | % | 10 | Khalil 2009 |
| LIC (Low-income country) River/canal water | % | 2.8 | Khalil 2009 |
| Sh. Sonnei survival on hands | hours | 3 | Hutchinson 1956 |
| Sh. Sonnei survival on lavatory seat | days | 17 | Hutchinson 1956 |
| Viability of S. Dysenteriae 110462 on cloth | hours | 8 | Hossain 2002 |
| Viability of S. Dysenteriae 14444 on cloth | hours | 24 | Hossain 2002 |
| Internal handle of public restroom | % | 3.3 | Na’was 2018 |
| Survival on cotton at 15C | days | 19-27 | Nakamura 2009 |
| Shigellosis and family member common use toilets | Frac. of cases | 4/7 | Nisar 2014 |
| Vibrio Cholera on LIC Latrine Floor | CFU/cm2 | 1-<5,000 | Gallandt 2020 |
| Vibrio Cholera on LIC Latrine door/wall | CFU/cm2 | 1-200 | Gallandt 2020 |

***** =** variable was not used to calculate risk in scenario

**Table S4. Concentration of protozoan cysts/oocysts and helminth ova on toilet surfaces**

| **Risk Parameter** | **Units** | **Value** | **Reference** |
| --- | --- | --- | --- |
| Helminth infections children using water cistern | % | 12.7 | IIechukwu (2010) |
| Prevalence of *Ascaris. lumbricoides* in Water Cistern children | % | 58 | IIechukwu (2010) |
| Prevalence of T. trichiura in Water Cistern children | % | 21 | IIechukwu (2010) |
| Prevalence of Hookworm in Water Cistern children | % | 21 | IIechukwu (2010) |
| Prevalence of A lumbricoides in closed latrines non pregnant women | % | 42.6 (95%CL: 30.7-54.6) | Nguyen 2006 |
| Prevalence of *Trichuris trichiura* in closed latrines non pregnant women | % | 21.9 (95% CL: 14.2-29.7) | Nguyen 2006 |
| Prevalence of Hookworm in closed latrines non pregnant women | % | 18.5 (95% CL: 12.1 - 24.9 ) | Nguyen 2006 |
| Latrine use hookworm infection concentration | eggs/g | 22 - 99 (odds ratio: 1.31 -1.27(0.55-3.31)) | Chongsuvivatwong (1996) |
| Helminth ova on Toilet seats | % | 37.5 | Wogu (2020) |
| Helminth ova on Toilet Flush handles | % | 17.5 | Wogu (2020) |
| Helminth ova on Restroom floor | % | 10 | Wogu (2020) |
| Helminth ova on Door handles | % | 17.5 | Wogu (2020) |

***** =** variable was not used to calculate risk in scenario
